# The Prevalence of Asthma among Iranian Children and Adolescent: A Systematic Review and Meta-Analysis

**DOI:** 10.1155/2021/6671870

**Published:** 2021-08-21

**Authors:** Neda Rahimian, Mohammad Aghajanpour, Leila Jouybari, Pedram Ataee, Asadollah Fathollahpour, Nasrin Lamuch-Deli, Wesam Kooti, Rasoul Nasiri Kalmarzi

**Affiliations:** ^1^Endocrine Research Center, Institute of Endocrinology and Metabolism, Iran University of Medical Sciences (IUMS), Tehran, Iran; ^2^ENT & Head and Neck Research Center and Department, Hazrat Rasoul Hospital, The Five Senses Institute, Iran University of Medical Sciences, Tehran, Iran; ^3^Nursing Research Center, Golestan University of Medical Sciences, Gorgan, Iran; ^4^Liver and Digestive Research Center, Research Institute for Health Development, Kurdistan University of Medical Sciences, Sanandaj, Iran; ^5^Department of Pediatrics, Faculty of Medicine, Kurdistan University of Medical Sciences, Sanandaj, Iran; ^6^Nutrition and Metabolic Diseases Research Center, Ahvaz Jundishapur University of Medical Sciences, Ahvaz, Iran; ^7^Lung Diseases & Allergy Research Center, Research Institute for Health Development, Kurdistan University of Medical Sciences, Sanandaj, Iran

## Abstract

**Background:**

Asthma is an important reason for hospitalization in children aged under five years. Information about the current status of asthma in Iranian children can help the Iranian health sector plan carefully and prevent asthma incidence by educating the families. The present systematic review and meta-analysis is aimed at estimating asthma prevalence in Iranian children and adolescents.

**Method:**

Data were found using keywords such as prevalence, epidemiology, asthma, adolescent, children, pediatrics, Iran in Web of Science, Scopus, PubMed, Cochrane, and Embase databases. Three national databases, including Magiran, Barakat Pharmed Co (Iran medex), and Scientific Information Databank (SID) were searched until 1 October 2020. Cross-sectional and original studies were included in the study, and then, quality assessment was done using the National Institutes of Health's Quality Assessment Tool for Observational Cohort and Cross-Sectional Studies. A pooled estimated prevalence of asthma was calculated using Der Simonian-Laird random model. Egger's test was used to evaluate publication bias. The data were analyzed using the STATA software version 16.

**Results:**

30 studies were selected and investigated. The prevalence of asthma in children and adolescents was 6% and 8%, and the prevalence in boys and girls was 9% and 8%, respectively. Among the asthma symptoms, wheezing had the most prevalence (17% in children and 19% in adolescents) and sleep disturbance had the lowest prevalence (6% in children and 6% in adolescents).

**Conclusion:**

The prevalence of asthma in Iranian children and adolescents is lower than in the world. Existing strategies should be pursued followed. Also, guidelines for asthma control and prevention should be considered in the future.

## 1. Background

Asthma is an airway inflammatory disease characterized by variable symptoms like chest tightness, breathlessness, wheeze, and cough. Asthma is one of the leading chronic respiratory diseases in children in the world. This disease is one of the main reasons for hospitalizations of children under five years old and has been increasing in recent years [1].

The estimations suggest that more than 300 million people are affected by asthma worldwide, and more than 400 million persons will experience asthma in the future. The prevalence of asthma in children varies according to geographical variation. However, the global prevalence is from 9.1% to 9.5% in children and from 9.1% to 10.4% in adolescents [2].

It seems that factors such as age, sex, economic status, genetics, and exposure to pollutants can affect asthma prevalence [3]. Asthma causes growth disturbance, increased health care costs, decreased quality of life, absence at school, etc. Implementation of proper strategies for recognizing the epidemiology of asthma and, consequently, appropriate treatment of the disease has effectively decreased disease burden [4].

The prevalence of asthma symptoms in children and adolescents increased worldwide, in recent years, particularly in low-middle income countries [5]. Many studies have been performed to estimate the prevalence of asthma in children and adolescents in various cities in Iran. Heidarnia et al. evaluated 19 studies between 1998 and 2003; all of them used ISSAC protocol. The lowest and the highest prevalence were reported in Kerman (2.7%) and Tehran (35.4%), respectively [6]. The overall prevalence of asthma was estimated to be 13.14%. In another meta-analysis, Ghaffari et al. evaluated 27 studies between 1992 and 2012. They reported asthma prevalence to be 2.7% and 3.5% in children aged 6-7 and 13-14 years, respectively [7]. These two meta-analyses showed a noticeable difference in the total asthma prevalence; also, there is no updated information about asthma status in Iranian children in recent years.

Information about the current status of asthma in Iranian children can help the Iranian health sector plan carefully and prevent asthma incidence by educating the families. It can also help neighboring countries to control the incidence of this disease because management of the health of this generation is important for any country. Due to Iran's geographical variety, the prevalence of this disease varies from region to region. As there is no updated comprehensive study to evaluate asthma prevalence in Iranian children and adolescents, the present systematic review and meta-analysis was performed to estimate asthma prevalence in children and adolescents in Iran.

## 2. Method and Material

The present study was performed based on Preferred Reporting Items for Systematic Reviews and Meta-Analyses (PRISMA) instruction [8] to examine asthma prevalence among children and adolescents in Iran.

### 2.1. Search Strategy

Data were found using keywords such as asthma, prevalence, epidemiology, children, pediatrics, adolescent, Iran in Scopus, PubMed, Web of Science, Cochrane and Embase databases.

For PubMed databases, this syntax was used: (Prevalence[Mesh] OR Prevalence[TIAB] OR Epidemiology[Mesh] OR Epidemiology[TIAB]) AND (Asthma[Mesh] OR Asthma∗[TIAB]) AND (Child[Mesh] OR Child∗[TIAB] OR “Adult Children”[Mesh] OR “Adult Children”[TIAB] OR Pediatrics[Mesh] OR Pediatrics∗[TIAB]) AND (Iran[Mesh] OR Iran∗[Text word]). Also, all the big cities searched separately to find the possible related studies. Three national databases, including Magiran, Barakat Pharmed Co (Iran medex), and Scientific Information Databank (SID) were searched until 1 October 2020. All articles published in internationally accredited journals, national conferences, and all available dissertations that attempted to assess the prevalence of asthma in different parts of the country were collected. All references were reviewed manually.

### 2.2. Inclusion Criteria

Studies that evaluated asthma prevalence in Iranian children were reviewed. The instrument used for data collection was ISSAC or SF-questionnaires, which study participants or their parents completed. Children were categorized as 0 to 10 years old, and those aged 11 to 19 were classified as adolescents. Cross-sectional and original studies were included in the study, and review articles or letter to editors was removed.

### 2.3. Exclusion Criteria

Unrelated topics, non-Iranian subjects, nonstandard questionnaires, incomplete data, and studies not defined the age group were excluded from the present study. Also, review articles and case-control studies were excluded.

### 2.4. Quality Assessment

In this step, the papers were independently evaluated by two authors. For quality assessment, we used the National Institutes of Health's Quality Assessment Tool for Observational Cohort and Cross-Sectional Studies, which resolve past versions' flaws. In this questionnaire, scoring is shown by a scale of “No,” “Yes,” “cannot be determined”, “Not Applicable,” and “Not reported” [9].

### 2.5. Data Extraction

The data extracted from all articles were used in the present study and qualified as a checklist. This checklist includes the author's name, type of study, year of publication, assessment tool, study location, sample size, age, gender, asthma prevalence, and associated risk factors.

### 2.6. Subgrouping the Outcome

In this study, the prevalence of asthma in children, adolescents, girls, and boys was considered the primary outcome. Other related information such as geographical area and asthma symptoms were considered as the secondary outcome.

### 2.7. Statistical Analysis

A pooled estimated prevalence of asthma was calculated using Der Simonian-Laird random model [10], and the results were reported by 95% confidence interval (CI). To examine the heterogeneity, *I*^2^ threshold was used [11]. Also, subgroup was done based on asthma symptoms and geographical areas. For finding the possible reasons of heterogeneity, metaregression was used based on the sample size and publication years. Sensitivity analysis was performed to ensure the stability of the results. Egger's test was used to evaluate publication bias [12]. The data were analyzed using the STATA software version 16.

## 3. Results

### 3.1. Search Results

**Figure 1:**
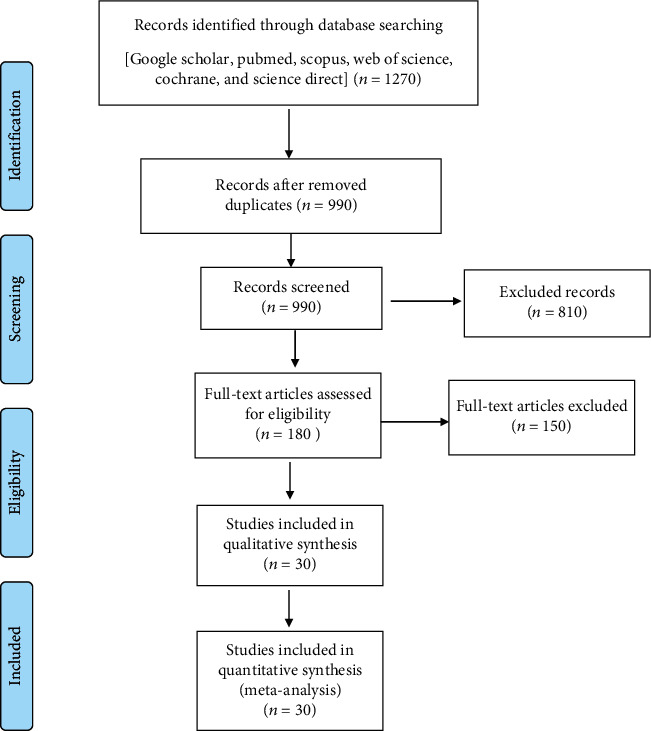
Flowchart describing the study design process.

In the first stage, 1270 articles were found; by reviewing the articles, 1090 duplicate and irrelevant articles with inadequate data and other exclusion factors were removed and finally, 30 articles were selected for this systematic review study (Figure 1).

### 3.2. Quality Assessment Results

This study used the National Institutes of Health Quality Assessment Tool for Observational Cohort and Cross-Sectional Studies. The studies' assessment showed that most of the studies had satisfactory quality and low-risk bias; however, most of them did not mention blinding of the outcome assessors. Most of the studies did not mention the research's timing in the title (Table 1).

### 3.3. Descriptive Results

Descriptive information of all studies was shown in Table 2. There are 30 studies in this table, of which the lowest prevalence of asthma in children was 2% in Masjedi et al.'s study in Tehran. The lowest prevalence of asthma in adolescents was 1% in the study of Hatami et al. in Bushehr. The highest prevalence of asthma in children was 32% in Tehran and 37% in adolescents. The majority of the study population was between 1,000 and 10,000. Only two studies had a population of less than 1,000. All studies use ISSAC questionnaire to gather the data. Most studies were performed in Tehran province (8 studies).

### 3.4. The Overall Prevalence of Asthma

**Figure 2:**
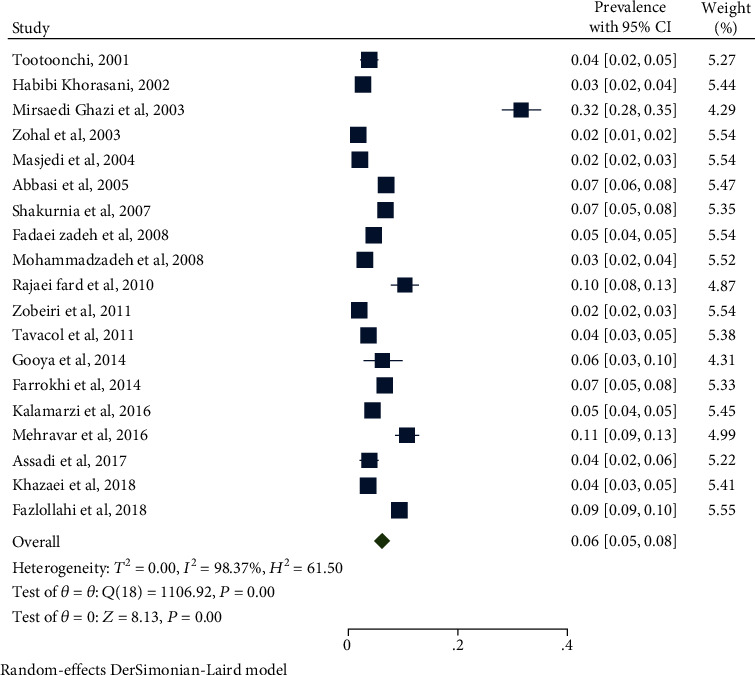
Prevalence of asthma in Iranian children (0-10).

**Figure 3:**
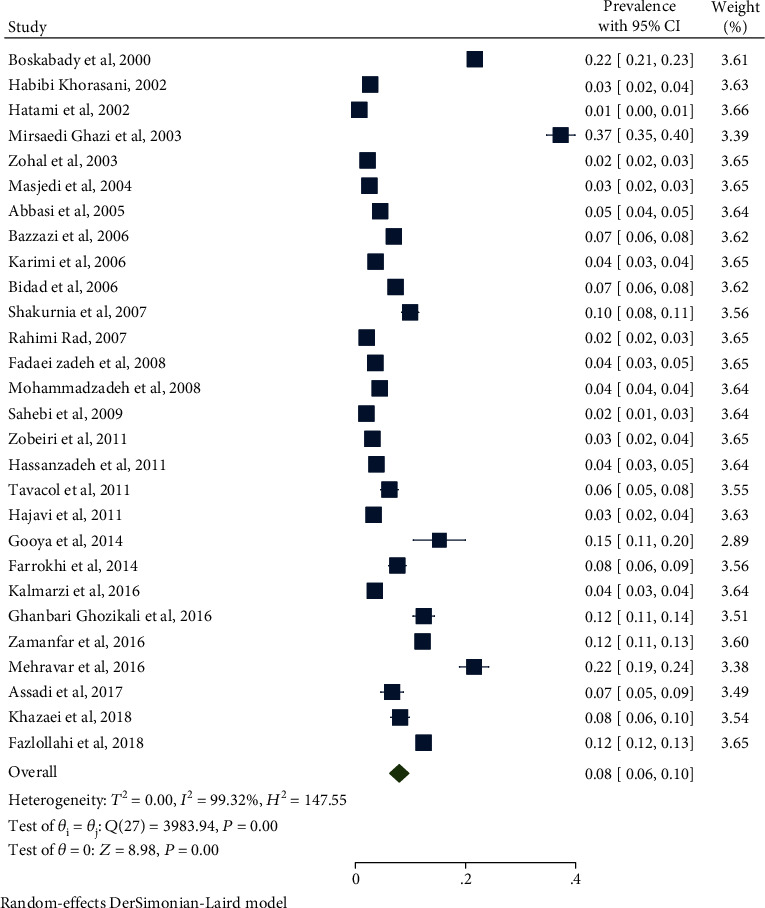
Prevalence of asthma in Iranian adolescent (11–19).

Table 3 shows the prevalence of asthma in the four groups of children, adolescents, boys, and girls in the selected articles. As shown in Table 3, the prevalence of asthma in children was 7% (95% CI: 5-9) with heterogeneity of 98.37% (Figure 2). The prevalence of asthma in adolescents was 9% (95% CI: 7-11) with heterogeneity of 99.32% (Figure 3). The prevalence of asthma in girl and boy were 8% (95% CI: 6-10) and 9% (95% CI: 7-11), respectively. The odds ratio for male/female and children/adolescents was 0.13 and 0.82, respectively. Sensitivity analysis showed that the results before and after this analysis did not change, and the findings were stable.

### 3.5. The Prevalence of Asthma Based on Geographical Areas

Iran's geographic areas were divided into five groups: North, South, Central, West, and East. The outbreak of asthma in the studies in each region was estimated as presented in Table 4. The highest prevalence of asthma in children and adolescents were in the Center and the East, respectively. The lowest prevalence of asthma was in children and adolescents in the West.

### 3.6. The Prevalence of Asthma Symptoms

As shown in Table 5, the outbreak of asthma symptoms and their effects were evaluated in this study. Among asthma symptoms in children and adolescents, wheezing had the most prevalence (17% in children and 19% in adolescents) and sleep disturbance had the lowest prevalence (6% in children and 6% in adolescents).

**Figure 4:**
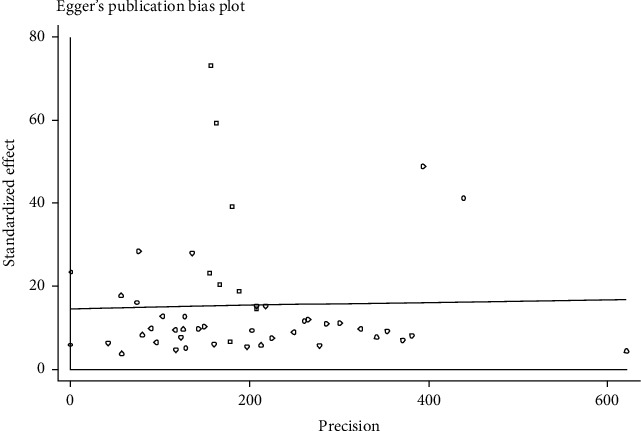
Publication bias in the studied researches (children and adolescent).

**Figure 5:**
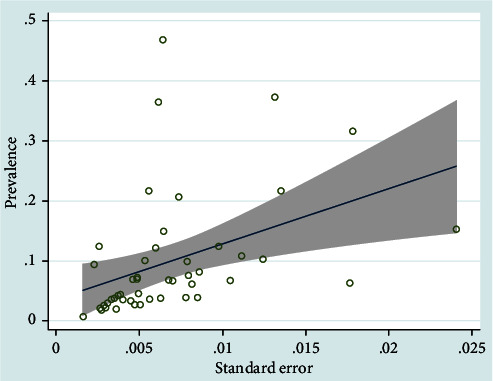
Metaregression for prevalence of asthma and years of studies (children and adolescent).

**Figure 6:**
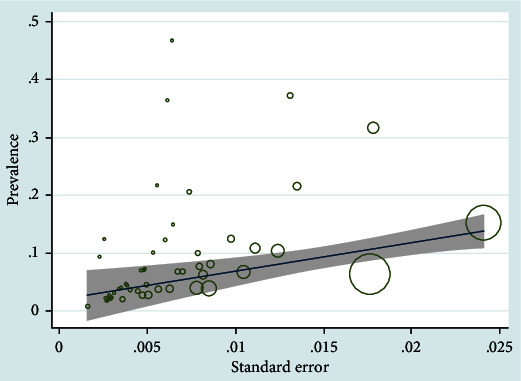
Metaregression for prevalence of asthma and sample size of studies (children and adolescent).

Publication bias of the asthma prevalence was significant (*P* < 0.001) (Figure 4). The prevalence of asthma by year (Figure 5) and sample size (Figure 6) was not significant (*P* > 0.05).

## 4. Discussion

The purpose of this systematic review and meta-analysis was to evaluate the prevalence of asthma among children and adolescents in Iran. This systematic review and meta-analysis investigated 30 studies from 2000 to 2020. The global outbreak of asthma has been increasing over the past decades. As a chronic disease that usually starts in early childhood, it imposes a heavy burden on these people's lives, caregivers, and the community. Despite significant progress in health care in the last decades, there is still ongoing disagreement between countries.

The prevalence of asthma in Iranian children and adolescents was 6% (95% CI: 5-8) and 8% (95% CI: 6-11). The global prevalence of asthma in children and adolescents has increased. Prevalence of ever asthma worldwide was 9.1% to 9.5% in children and 9.1% to 10.4% in adolescents [2]. The prevalence of asthma in children was reported to be 10.6%, 10.1%, 5.35%, 7.4%, 23.8%, and 10.7% in Oman (2016), Brazil (2019), India (2016), Thailand (2018), Costa Rica (2019), and Northern Portugal (2016), respectively. The prevalence of asthma in adolescents was 19.8%, 6.05%, 6.1%, and 25.9% in Oman, India, Thailand, and Costa Rica, respectively [13–18]. The prevalence of asthma in Iran in the present study is lower than the reported value in other countries (such as Brazil, Oman, Costa Rica, and Portugal), but it was higher than Indian children and adolescents in Thailand and India. Many types of researches in Iran have examined the prevalence of asthma, leading to control of asthma incidence. Differences in variables such as climate, lifestyle, nutrition, ethnicity, and cultures in Iran compared to other countries can also affect asthma prevalence.

Many studies have been performed to estimate the prevalence of asthma in children and adolescents in various cities in Iran. Heidarnia et al. evaluated 19 studies between 1998 and 2003, and the overall prevalence of asthma was estimated to be 13.14%, which was higher than our study [6]. In another meta-analysis, Ghaffari et al. evaluated 27 studies between 1992 and 2012. They reported asthma prevalence to be 2.7% and 3.5% in children aged 6-7 and 13-14 years, respectively [7]. In Hassanzadeh et al.'s study, just guidance school children were evaluated between 1997 and 2009. The overall outbreak was 3.9% [19]. Varmaghani et al. evaluated 10 studies between 1990 and 2015; they reported the asthma prevalence to be 8.80% [20]. Compared with the present results, these reports showed that asthma prevalence increased in recent years in Iran.

According to the present systematic review and meta-analysis, the prevalence of asthma in adolescents was higher than in children (OR = 0.82, CI: 0.64-1.06, and *P* < 0.001). This was consistent with several studies, such as Soto-Martínez et al., Al-Herz, de Oliveira et al., and Singh et al. [13, 17]. This may be because adolescents spend more time outside the home and are more exposed to contaminants and other allergens and pollen. This finding may also be due to changes during puberty, since hormonal changes at this age can affect the incidence and severity of asthma [3]. Smoking as well is more common in this age group and can increase the prevalence of asthma. Other causes of increased asthma prevalence in adolescents could include increased consumption of foods and drinks containing preservatives.

In this systematic review and meta-analysis, the prevalence of asthma was found to be higher in boys (9%, 95% CI: 7-11) than in girls (8%, 95% CI: 6-10) (OR = 0.13, CI: -0.04-0.30, and *P* < 0.001). Several studies were similar to the present study, such as the study of Chinratanapisit et al. in Bangkok, and other studies in Turkey and Korea [16, 21, 22]. Also in Japan, the prevalence of asthma was higher in boys (6%) than girls (4%) [23]. These gender differences might be ascribed to a narrower airways caliber in males than females in early life due to different hormonal factors. Other reasons include physical and social differences. Since smoking is more prevalent in boys than in girls, it can influence this disease [24].

The other causes of the difference in asthma prevalence include climate dispersal in a different region of Iran. In the present systematic review and meta-analysis, among region subgroups, the highest and lowest prevalence of asthma in children was 8% and 3% in the Center and the West of Iran, respectively. Also, the highest and lowest prevalence of asthma in adolescent were 13% in the East and 4% in the West of Iran. As a result, the central region's prevalence was higher, which may be due to air pollution and contaminants in central populated cities such as Tehran. According to Lashanizand and Gholamrezaie's study, atmospheric conditions directly and indirectly affect the prevalence of asthma, and ambient moisture reduces asthma attacks, according to the present study. It can be concluded that the northern and southern regions of the sea margin have suitable ambient humidity, which results in a lower prevalence of asthma in these regions [25]. In addition to climate change, air pollution and vehicle overcrowding in crowded cities, diet in different areas, and genetic changes can also affect asthma prevalence. Also, scattering vegetation in different cities and exposure to pollen in different seasons can affect asthma prevalence. As shown in the study of Zhang et al., SO_2_, relative humidity and sunshine are associated with asthma prevalence [26].

Descriptive information including wheezing, dry cough, sleep disturbance, wheezing attacks, and exercise wheeze was estimated in the present study. The prevalence of asthma symptoms in children and adolescents worldwide increased from 11.1% to 11.6% and from 13.2% to 13.7%, respectively, between ISAAC phase I and phase III. According to the present systematic review and meta-analysis, wheezing prevalence was higher in Iranian adolescents than in children (17% in children and 19% in adolescents). In a study in Thailand (Southeast Asia), wheezing was reported 14.6% in children and 12.5% in adolescents [16]. A study by Mallol et al. reported the prevalence of wheezing from 11.11% in 1994 to 13.4% in 2015 among adolescents [3]. It was 35.1% in children and 35.4% in adolescent in Costa Rica [17]. The prevalence of wheezing in Thailand was lower than in the present study but higher in Brazil and Costa Rica than in the present study. It was higher in adolescents than children in the present study; this is in line with Costa Rica study. The prevalence of dry cough was higher in adolescents than in children (11% in children and 17% in adolescents). The prevalence of dry cough in a study in Thailand (24.2% in children and 29.9 in adolescents) was similar to the present study; however, the result of Costa Rica study (28.5% in children and 24.4% in adolescent) was different with our result [16, 17]. In Mallol et al.'s study (2015), it was reported to be 33.1% in adolescents. The prevalence of dry cough in the present study was lower than studies done in Thailand, Costa Rica, and South-Santiago. The prevalence of exercise-induced wheezing was higher in adolescents than in children (7% in children and 16% in adolescents). In Chinratanapisit et al.'s study in Thailand, it was 3% in children and 14.8% in adolescents [16], which was higher than the amount reported in the present study.

The prevalence of wheezing attacks was significantly higher in adolescents than in children, and this finding is in line with a study in Thailand [16]. Having stress during this period of life is more than childhood and can affect asthma wheezing attacks. The prevalence of wheezing attacks in the present study was lower than in children and higher than in adolescents in Thailand. As shown, the prevalence of asthma symptom in adolescent was higher than children. The reasons can also be attributed to the increased prevalence of asthma in adolescents than children.

Publication bias of the asthma prevalence was significant. The quality of the papers affects the bias. Most of the studies did not mention blinding of the outcome assessors. Also, most of them did not mention the research's timing in the title. The results may affect by some limitation in the researches. Also, heterogeneity was encountered perhaps due to various center settings, populations enrolled, etc. In some parts of the country, we do not find any research; few studies have been done in other parts. This limitation may affect the results and cause publication bias. This study was not registered in PROSPERO.

## 5. Conclusion

Overall, the present study showed that asthma prevalence in Iranian children and adolescents is lower than in regions in the world. Given that the disease affects the quality of life of the studied age group and the quality of their education and economic situation, the existing strategies should be pursued as well, on the provision of medicines and the cost of treatment. Also, guidelines for asthma control should be considered in the future, such as providing asthma medicines throughout the country (especially villages), measures to reduce air pollution, and training parents to quit smoking, alternatively, smoking in a place far away from their children.

## Figures and Tables

**Table 1:** The score of the studied based on National Institutes of Health Quality Assessment Tool for Observational Cohort and Cross-Sectional Studies.

Author/studies	Q1	Q2	Q3	Q4	Q5	Q6	Q7	Q8	Q9	Q10	Q11	Q12	Q13	Q14
Boskabady and Karimian	×	✓	✓	✓	✓	NA	✓	✓	✓	✓	✓	NR	✓	✓
Tootoonchi	×	✓	✓	✓	✓	NA	CD	×	✓	NA	✓	NR	✓	✓
Hatami et al.	✓	✓	✓	✓	NR	NR	✓	✓	✓	NA	✓	CD	✓	✓
Habibi et al.	✓	✓	✓	✓	✓	NA	✓	✓	✓	NA	✓	NR	✓	✓
Zohal and Hasheminasab	✓	✓	✓	✓	✓	NA	NR	✓	✓	NA	✓	NR	✓	✓
Ghazi et al.	✓	✓	✓	✓	✓	NA	✓	✓	✓	NA	✓	NR	✓	✓
Masjedi et al.	×	✓	✓	✓	✓	NA	✓	✓	✓	NA	✓	NR	✓	✓
Abbasi	×	✓	✓	✓	✓	NA	✓	✓	✓	NA	✓	NR	✓	✓
Bazzazi et al.	×	✓	✓	✓	✓	NA	NR	✓	✓	NA	✓	NR	✓	✓
Karimi et al.	×	✓	✓	✓	✓	NA	✓	✓	✓	NA	✓	NR	✓	✓
Bidad et al.	✓	NR	×	✓	✓	NA	✓	×	✓	NA	✓	NR	✓	✓
Shakurnia et al.	×	✓	✓	✓	✓	NA	NR	✓	✓	NA	✓	NR	✓	✓
Rahimi Rad et al.	×	✓	✓	✓	✓	NA	✓	✓	✓	NA	✓	NR	✓	✓
Fadaeizadeh et al.	✓	✓	✓	✓	✓	NA	✓	✓	✓	NA	✓	NR	✓	✓
Mohammadzadeh et al.	×	✓	✓	✓	✓	NA	✓	✓	✓	NA	✓	NR	✓	✓
Sahebi and Shabestray	×	✓	✓	✓	✓	NA	✓	✓	✓	NA	✓	NR	✓	✓
Rajaeifard et al.	✓	✓	✓	✓	✓	NA	NR	✓	✓	NA	✓	NR	✓	✓
Zobeiri	✓	✓	✓	✓	✓	NA	✓	✓	✓	NA	✓	NR	✓	✓
Tavacol et al.	×	✓	✓	✓	✓	NA	✓	✓	✓	NA	✓	NR	✓	✓
Hassanzadeh et al.	✓	✓	✓	✓	✓	NA	✓	CD	✓	NA	×	NR	✓	✓
Hajavi et al.	✓	✓	✓	✓	✓	NA	✓	✓	✓	NA	✓	NR	✓	✓
Gooya et al.	✓	✓	✓	✓	✓	NA	✓	✓	✓	NA	✓	NR	✓	✓
Farrokhi et al.	✓	✓	✓	✓	✓	NA	✓	✓	✓	NA	✓	NR	✓	✓
Nasiri Kalmarzi et al.	✓	✓	✓	✓	✓	NA	✓	✓	✓	NA	✓	NR	✓	✓
Ghozikali et al.	✓	✓	✓	✓	✓	NA	✓	✓	✓	NA	✓	NR	✓	✓
Zamanfar et al.	✓	✓	✓	✓	✓	NA	✓	✓	✓	NA	✓	NR	✓	✓
Mehravar et al.	✓	✓	✓	✓	✓	NA	✓	✓	✓	NA	✓	NR	✓	✓
Assadi et al.	✓	✓	✓	✓	✓	NA	✓	✓	✓	NA	✓	NR	✓	✓
Khazaei et al.	×	✓	✓	✓	✓	NA	✓	✓	✓	NA	✓	NR	✓	✓
Fazlollahi et al.	✓	✓	✓	✓	✓	NA	✓	✓	✓	NA	✓	NR	✓	✓

**Table 2:** Characteristics of all eligible asthma prevalence studies in children and adolescents in Iran.

Author name	Year	City (province)	Method	Prevalence in children		Prevalence in adolescent		Prevalence in girl		Prevalence in boy		References
				%	*n*	%	*n*	%	*n*	%	*n*	
Boskabady and Karimian	2000	Mashhad (Khorasan Razavi)	ISSAC	_	_	22	1201	25	618	19	583	[[27]]
Tootoonchi	2001	Tehran (Tehran)	ISSAC	4	24	_	_	3	9	5	15	[[28]]
Hatami et al.	2002	Bushehr (Bushehr)	ISSAC	_	_	1	19	_	_	_	_	[[29]]
Habibi et al.	2002	Kerman (Kerman)	ISSAC	3	28	3	32	2	27	3	33	[[30]]
Zohal and Hasheminasab	2003	Ghazvin (Ghazvin)	ISSAC	2	47	2	57	2	45	2	59	[[31]]
Ghazi et al.	2003	Tehran (Tehran)	ISSAC	32	215	37	507	34	343	37	379	[[32]]
Masjedi et al.	2004	Tehran (Tehran)	ISSAC	2	64	3	80	3	80	2	64	[[33]]
Abbasi	2005	Rasht (Gilan)	ISSAC	7	213	5	136	5	144	7	205	[[34]]
Bazzazi et al.	2006	Gorgan (Golestan)	ISSAC	_	_	7	196	6	86	8	110	[[35]]
Karimi et al.	2006	Yazd (Yazd)	ISSAC	_	_	4	118	_	_	_	_	[[36]]
Bidad et al.	2006	Tehran (Tehran)	ISSAC	_	_	7	212	6	99	9	106	[[37]]
Shakurnia et al.	2007	Ahvaz (Khuzestan)	ISSAC	7	96	10	144	_	_	_	_	[[38]]
Rahimi Rad et al.	2007	Urmia (West Azarbaijan)	ISSAC	_	_	2	62	1	_	3	_	[[39]]
Fadaeizadeh et al.	2008	Tehran, Rasht (Tehran, Gilan)	ISSAC	5	283	4	222	_	_	_	_	[[40]]
Mohammadzadeh et al.	2008	Babol (Mazandaran)	ISSAC	3	91	4	128	4	119	5	141	[[41]]
Sahebi and Shabestray	2009	Tabriz (East Azarbaijan)	ISSAC	_	_	2	30	_	_	_	_	[[42]]
Rajaeifard et al.	2010	Yasuj (Kohgiluyeh and Boyer-Ahmad)	ISSAC	10	62	_	_	7	23	13	39	[[43]]
Zobeiri	2011	Kermanshah (Kermanshah)	ISSAC	2	63	3	99	4	110	2	52	[[44]]
Tavacol et al.	2011	Ahvaz (Khuzestan)	ISSAC	4	35	6	54	_	_	_	_	[[45]]
Hassanzadeh et al.	2011	Shiraz (Fars)	ISSAC	_	_	4	115	3	51	4	64	[[46]]
Hajavi et al.	2011	Gonabad (Khorasan Razavi)	ISSAC	_	_	3	54	_	_	_	_	[[47]]
Gooya et al.	2014	Bushehr (Bushehr)	ISSAC	6	11	15	34	13	38	6	7	[[48]]
Farrokhi et al.	2014	Bushehr (Bushehr)	ISSAC	7	86	8	85	_	_	_	_	[[49]]
Nasiri Kalmarzi et al.	2016	Kurdistan	ISSAC	5	80	4	75	_	_	_	_	[[50]]
Ghozikali et al.	2016	Tabriz (East Azarbaijan)	ISSAC	_	_	12	142	_	_	12	142	[[51]]
Zamanfar et al.	2016	Mazandaran	ISSAC	_	_	12	362	9	140	16	222	[[52]]
Mehravar et al.	2016	Golestan	ISSAC	11	84	22	200	18	156	15	128	[[53]]
Assadi et al.	2017	Bushehr (Bushehr)	ISSAC	4	20	7	38	5	25	5	33	[[54]]
Khazaei et al.	2018	Tehran (Tehran)	ISSAC	4	40	8	82	6	63	6	59	[[55]]
Fazlollahi et al.	2018	Tehran (Tehran)	ISSAC	9	1543	12	2090	10	_	12	_	[[56]]

**Table 3:** Overall results of the prevalence of asthma in Iranian children and adolescent.

Item	Prevalence	*P* value	*I* ^2^	OR	Number of study
Children	6% (5–8)	<.001	98.37%	0.82 (0.64-1.06)	19
Adolescent	8% (6–11)	<.001	99.32%		28
Girl	8% (6–10)	<.001	99.02%	0.13 (-0.04-0.30)	20
Boy	9% (7–11)	<.001	99.18%		21

**Table 4:** The prevalence of asthma by geographical area of Iran.

Region		Studies	Sample		Heterogeneity	
			All	%	I2	*P* value
Children	Central	7	30425	8	99.30%	<0.001
	North	3	6881	7	97.64%	<0.001
	South	7	5955	6	79.62%	<0.001
	East	_	_	_	_	_
	West	2	4823	3	94.82%	<0.001

Adolescent	Central	8	37130	9	99.58%	<0.001
	North	5	12605	10	98.53%	<0.001
	South	8	11175	6	97.83%	<0.001
	East	2	7160	13	99.85%	<0.001
	West	5	10954	4	99.51%	<0.001

**Table 5:** Asthma symptoms in Iranian children and adolescent.

Item		Prevalence	*P* value	*I* ^2^	OR	Number of study
Wheezing	Children	17% (13–20)	<.001	98.65%	0.78 (0.64-0.95)	16
	Adolescent	19% (15–23)	<.001	99.58%		23
	Boy	19% (15–23)	<.001	98.83%	0.04 (-0.18-0.26)	16
	Girl	18% (15–21)	<.001	98.61%		18

Dry cough	Children	11% (9–14)	<.001	98.47%	0.61 (0.46-0.80)	15
	Adolescent	17% (15–20)	<.001	98.85%		21
	Boy	18% (15–21)	<.001	98.03%	0.17 (-0.01-0.35)	14
	Girl	15% (12–17)	<.001	98.41%		14

Exercise wheezing	Children	7% (5–9)	<.001	99.03%	0.33 (0.26-0.41)	13
	Adolescent	16% (13–19)	<.001	98.94%		18
	Boy	17% (12–22)	<.001	98.73%	4.31 (3.79-4.82)	13
	Girl	12% (9–14)	<.001	99.55%		13

Wheezing attack	Children	13% (9–17)	<.001	98.43%	0.82 (0.78-0.86)	5
	Adolescent	14% (9–18)	<.001	99.25%		7
	Boy	15% (7–22)	<.001	98.87%	0.15 (0.06-0.24)	4
	Girl	14% (7–21)	<.001	98.85%		4

Sleep disturbance	Children	6% (4–7)	<.001	96.28%	1.03 (0.93-1.14)	11
	Adolescent	6% (4–7)	<.001	98.02%		14
	Boy	8% (6–11)	<.001	98.02%	0.06 (-0.05-0.18)	7
	Girl	7% (5–9)	<.001	98.48%		7

## Data Availability

All data generated or analyzed during this study are included in this published article.
